# Concussion in soccer: a comprehensive review of the literature

**DOI:** 10.2217/cnc-2020-0004

**Published:** 2020-07-01

**Authors:** James Mooney, Mitchell Self, Karim ReFaey, Galal Elsayed, Gustavo Chagoya, Joshua D Bernstock, James M Johnston

**Affiliations:** 1 Department of Neurosurgery, University of Alabama at Birmingham, 1813 6th Ave S #516, Birmingham, AL 35233, USA; 2 Department of Neurosurgery, Mayo Clinic, 4500 San Pablo Rd S, Jacksonville, FL 32224, USA; 3 Department of Neurosurgery, Brigham & Women’s Hospital, Harvard Medical School, 75 Francis St, Boston, MA 02115, USA

**Keywords:** football, repetitive subconcussive head impact, soccer, sport injuries, sport-related concussion, traumatic brain injury

## Abstract

Sports-related concussion has been examined extensively in collision sports such as football and hockey. However, historically, lower-risk contact sports such as soccer have only more recently garnered increased attention. Here, we review articles examining the epidemiology, injury mechanisms, sex differences, as well as the neurochemical, neurostructural and neurocognitive changes associated with soccer-related concussion. From 436 titles and abstracts, 121 full texts were reviewed with a total of 64 articles identified for inclusion. Concussion rates are higher during competitions and in female athletes with purposeful heading rarely resulting in concussion. Given a lack of high-level studies examining sports-related concussion in soccer, clinicians and scientists must focus research efforts on large-scale data gathering and development of improved technologies to better detect and understand concussion.

Sports-related concussion (SRC) and mild traumatic brain injury (TBI) have become topics of major public health interest with the US Centers for Disease Control and Prevention (GA, USA) declaring that SRC is reaching “*epidemic levels*” and deserves further investigation [1–3]. Significant focus has been given to SRC in American football and ice hockey, with soccer only garnering increased attention within the past decade due to its worldwide popularity. Currently, soccer is the most popular and fastest-growing sport worldwide [4]. While soccer has historically been considered a lower risk contact sport, the American Academy of Pediatrics (IL, USA) has recently ranked soccer equivalent to American football and ice hockey, with a comparable frequency of head injury [5,6]. Head trauma in soccer is frequently underdiagnosed with potential consequences neglected due to heterogeneous and often mild symptoms. The consensus statement adopted by the Federation Internationale de Football Association (FIFA; Zürich, Switzerland) from the 2012 and 2016 International Conference on Concussion in Sport, defines SRC as an injury that represents “*immediate and transient symptoms of traumatic brain injury (TBI)*” [7]. The consensus states that players in question must be immediately evaluated for any feature of concussion with a side-line diagnostic assessment evaluating state of consciousness, orientation, cranial nerve function and balance, and then be withdrawn from play if concussion has occurred [7,8]. Various diagnostic tools including the Sport Concussion Assessment Tool (SCAT) have been developed that has most recently been updated to the SCAT-5.

This comprehensive review provides a summary of the literature examining the epidemiology, mechanisms of injury, sex differences, short- and long-term neurochemical, neurostructural and neurocognitive consequences, as well as recommendations for prevention of SRC in soccer.

## Methods

### Search strategy

**Figure 1.**
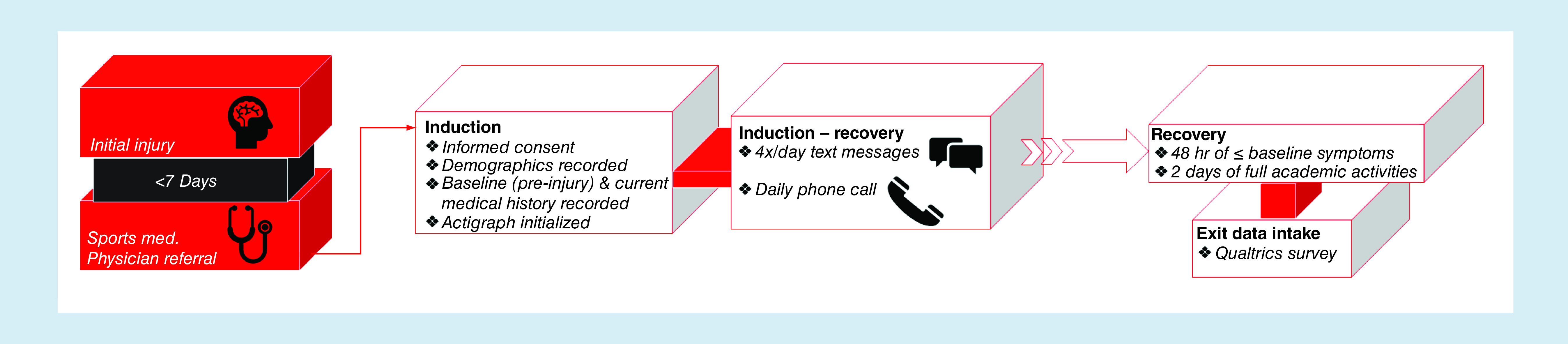
Flowchart demonstrating the review process.

A literature search was developed by the primary author (J Mooney) and conducted in May 2020, using PubMed and Medline, limited to English language. Search phrases including ‘soccer; concussion; mild TBI; repetitive subconcussive head impact (RSHI); sex differences; repetitive heading; neurochemistry; chronic traumatic encephalopathy (CTE); neuropsychiatric; epidemiology; neurocognitive; injury mechanisms and prevention’ were combined using the operators ‘AND’ and ‘OR’. Titles and abstracts were screened by the primary author, and independent authors reviewed relevant full-text articles. Studies were grouped into categories based on specific areas of focus. 121 full texts were initially screened for inclusion based on the relevance of the title and abstract. Bibliographies of full texts were surveyed for additional pertinent studies. Ultimately, 64 unique articles were chosen for final inclusion. Figure 1 demonstrates the study selection process. Articles divided by category and study type are shown in Table 1. Several articles were included in multiple categories if parts of the articles pertained to different categories. Studies utilized in several categories are bolded in Table 4.

**Table 1.** Studies included in review by category and study type.

		Study type						
		All	Retrospective	Prospective	Lit review	Descriptive epidemiological	Cross-sectional	RCT
Category	Epidemiology	7	1	1	3	2	–	–
	Epidemiology/injury mechanisms	15	3	4	2	6	–	–
	Sex differences	10	1	3	–	3	3	–
	Biochemical/structural/CTE	20	4	12	2	–	2	–
	Neuropsychiatric	19	–	16	1	–	2	–
	Prevention	9	–	3	3	–	2	1
	Total	80^†^	9	39	11	11	9	1

† Several studies were included in multiple categories.

RCT: Randomized control trial.

### Eligibility criteria

Manual review of all articles was carried out for relevance by the primary author (J Mooney) and approved by all study authors. Using the criteria listed below, eligible articles were selected for full review.

### Inclusion criteria

Included articles were published in the English language and included literature reviews, retrospective, prospective, descriptive, cross-sectional and randomized control trials containing greater than five soccer athletes.

### Exclusion criteria

Articles examining sports other than soccer were excluded or only data examining soccer were extracted from those studies. Case reports and case series were excluded.

### Data extraction

Data relating to epidemiology, mechanisms, sex differences, neurochemical/structural/psychiatric, CTE and prevention were extracted from studies examining soccer-related concussion. Level of evidence and study types for the various categories is reported in Tables 1–3.

**Table 2.** Studies included examining injury prevention.

Study	(year)	Players/ studies/ events	Study type	Level of evidence	Study name	Summary/key points	Ref.
Quintero *et al*.	(2019)	Five players	Prospective	II	Reducing risk of head injury in youth soccer: an extension of behavioral skills training for heading	Behavior skills training is an acceptable form of training and helped players improve correct heading from baseline	[91]
McGuine *et al*.^†^	(2019)	2766 players	RCT	I	Does soccer headgear reduce the incidence of sport-related concussion? A cluster, randomized controlled trial of adolescent athletes	Headgear did not reduce incidence or severity of sports-related concussion in high school soccer players	[88]
Press *et al*.	(2017)	26 players	Prospective	III	Quantifying head impact exposure in collegiate women’s soccer	Users of head impact data must exercise caution when interpreting on-field head impact sensor data	[97]
Catenaccio *et al*.	(2016)	48 players	Prospective	III	Validation and calibration of HeadCount, a self-report measure for quantifying heading exposure in soccer players	HeadCount can be used to index exposure in population studies and, once generalizable safe exposure thresholds have been delineated, could be widely disseminated to monitor exposure and minimize risk	[98]
Caccese *et al*.	(2016)	18 studies	Literature review	III	Minimizing head acceleration in soccer: a review of the literature	Risk of concussive impacts may be lessened through the use of headgear, but headgear may also cause athletes to play more recklessly because they feel a sense of increased security	[92]
Faude *et al*.	(2013)	32 studies	Literature review	III	Football injuries in children and adolescent players: are there clues for prevention?	Areas of relevance for injury prevention: contact injuries during matches; high number of fractures in younger players; and influence of maturation status and growth spurts	[13]
Niedfeldt *et al*.	(2011)	NA	Literature review	III	Head injuries, heading and the use of headgear in soccer	Headgear has not been shown to be effective in reducing ball impact but may be helpful in reducing the force of nonball-related impacts to the head	[89]
Naunheim *et al*.	(2003)	NA	Cross-sectional study	III	Does soccer headgear attenuate the impact when heading a soccer ball?	Currently available headgear for soccer heading shows little ability to attenuate impact during simulated soccer heading	[87]
Babbs *et al*.	(2001)	NA	Cross-sectional study	III	Biomechanics of heading a soccer ball: implications for player safety	Heading is usually safe but occasionally dangerous, depending on key characteristics of both the player and the ball	[94]

† Studies of considerable interest.

NA: Not available; RCT: Randomized control trial.

**Table 3.** Studies included examining epidemiology/mechanisms of injury.

Study	(year)	Players/ studies/ events	Study type	Level of evidence	Study name	Summary/key points	Ref.
Reynolds *et al.*	(2017)	21 players	Descriptive epidemiological	IV	Effects of sex and event type on head impact in collegiate soccer	Soccer games resulted in more cumulative head impacts than practices	[29]
Kontos *et al*.	(2017)	28 studies	Systematic review and meta-analysis	I	Systematic review and meta-analysis of the effects of football heading	No overall adverse effect for heading a football	[14]
Khodaee *et al*.	(2017)	6154 soccer injuries	Descriptive epidemiological	IV	Nine-year study of US high school soccer injuries: data from a national sports injury surveillance program	Injury rates vary by sex, type of exposure. Injury patterns similar	[17]
Zuckerman *et al*.	(2016)	180 athletes (47 soccer)	Retrospective cohort	II	Mechanisms of injury as a diagnostic predictor of sport-related concussion severity in football, basketball and soccer: results from a regional concussion registry	Challenging a player, fighting for a loose ball and heading (not necessarily with ball contact) were proportionally the most common mechanisms of sports-related concussion in soccer	[28]
Comstock *et al*.^†^	(2015)	627 concussions	Retrospective	III	An evidence-based discussion of heading the ball and concussions in high school soccer	Athlete–athlete contact most frequent mechanism of concussion	[15]
Herrero *et al*.	(2014)	15,243 soccer injuries	Descriptive epidemiological	IV	Injuries among Spanish male amateur soccer players: a retrospective population study	Risk of injury in amateur soccer is lower than that previously reported in professional players	[34]
Nilsson *et al*.	(2013)	136 head and neck injuries	Prospective cohort	II	Head and neck injuries in professional soccer	Head and neck injuries were relatively uncommon in professional soccer. Defender was the playing position most at risk	[23]
Faude *et al*.	(2013)	32 studies	Literature review	III	Football injuries in children and adolescent players: are there clues for prevention?	Strains, sprains and contusions of lower extremities most common injury types	[13]
Marar *et al*.^‡^	(2012)	1936 concussions	Descriptive epidemiological	IV	Epidemiology of concussions among US high school athletes in 20 sports	Player-to-player and player-to-surface contact most common injury mechanisms in soccer	[16]
Gessel *et al*.	(2007)	396 concussions	Descriptive epidemiological	IV	Concussions among US high school and collegiate athletes	Concussion rates highest in football and soccer	[30]
Delaney *et al*.	(2006)	69 concussions in 60 athletes	Prospective cohort	II	Mechanisms of injury for concussions in university football, ice hockey and soccer: a pilot study	Hit to side/temporal area of head most associated with concussion in soccer	[31]
Faude *et al*.	(2006)	143 female players	Prospective cohort	II	Risk factors for injuries in elite female soccer players	Injury incidence significantly higher in defenders	[32]
Andersen *et al*.	(2004)	192 incidents, 17 head injuries	Descriptive epidemiological	IV	Mechanisms of head injuries in elite football	Elbow-to-head, followed by head-to-head contact were most frequent injury mechanisms	[9]
Delaney *et al*.^‡^	(2002)	240 players	Retrospective survey	III	Concussions among university football and soccer players	History of concussion increases odds of future concussion in soccer players	[11]
Boden *et al*.	(1998)	29 concussions, 26 athletes	Prospective cohort	II	Concussion incidence in elite college soccer players	Concussions more common in soccer than anticipated	[18]

† Studies of considerable interest.

‡ Studies of interest.

**Table 4.** Studies included in review (sex differences, neuropsychiatric, biochemical/structural/chronic traumatic encephalopathy).

Sex differences (n = 10)	Neuropsychiatric (n = 19)	Biochemical/structural/CTE (n = 20)	Ref.
Caccese *et al.* (2018)^‡^	Levitch *et al*. (2018)	Mackay *et al.* (2019)^†^	[40,64,74]
**Reynolds *et al.* (2017)**	**Pearce (2016)**	Wallace *et al.* (2018)	[29,57,67]
Chandran *et al*. (2017)	Forbes *et al.* (2016)	Svaldi *et al.* (2018)	[38,53,69]
Bretzin *et al*. (2017)	Di Virgillo *et al*. (2016)	Sadrameli *et al*. (2018)	[39,52,72]
**Covassin *et al*. (2013)**	**Koerte *et al*. (2015)**	Myer *et al.* (2019)	[42,46,48]
Zuckerman *et al.* (2012)	**Maher *et al*. (2014)**	Tarnutzer *et al.* (2017)	[43,44,75]
**Kontos *et al*. (2011)**	**Lipton *et al*. (2013)**	Ling *et al*. (2017)	[45,47,71]
Colvin *et al*. (2009)	**Covassin *et al*. (2013)**^‡^	Castro *et al*. (2017)	[41,42,99]
Tierney *et al*. (2008)	Rieder *et al.* (2011)	**Pearce (2016)**	[67,100,101]
Kraus *et al.* (1996)	**Kontos *et al*. (2011)**	Koerte *et al.* (2015)	[63,71,102]
	Kaminski *et al.* (2007)	Riley *et al.* (2015)	[65,103]
	Straume-Naesheim *et al*. (2005)	**Koerte *et al*. (2015)**	[48,70]
	Stephens *et al*. (2005)	Dorminy *et al*. (2015)	[55,104]
	Witol *et al.* (2003)	Bieniek *et al*. (2015)	[58,105]
	Webbe *et al.* (2003)	**Maher *et al*. (2014)**	[73,75]
	Matser *et al*. (2001)	**Lipton *et al*. (2013)**	[47,106]
	Putukian *et al*. (2000)	McKee *et al*. (2009)	[62,107]
	Matser *et al.* (1999)	Zetterberg *et al*. (2007)	[108,109]
	Matser *et al.* (1998)	Mussack *et al*. (2003)	[56,83]
		Geddes *et al*. (1999)	[59]

Bolded studies appear in multiple categories.

† Studies of considerable interest.

‡ Studies of interest.

CTE: Chronic traumatic encephalopathy.

## Results

### Epidemiology

4 to 22% of all soccer injuries are head/neck injuries with a reported incidence of 1.7 injuries per 1000 playing hours [9]. The incidence of concussions has been estimated at 0.5 injuries per 1000 playing hours; however, the accuracy of this estimate is difficult to determine given inconsistencies in the interpretation and reporting of concussions [9–11].

Over 80% of all soccer-related injuries reported in the USA occur among those under the age of 25 [12]. Faude *et al*. examined injuries to child and adolescent soccer players (aged 5–19 years) and reported that 5% of all injuries in these age groups were to the head [13]. In a more recent online survey of 8104 youth soccer teams including 101,699 players aged 7–14 years, the overall reported concussion incidence rate was 0.85/1000 athlete exposures (AEs) with concussions 5.7-times more likely to occur in games (1.73/1000 AEs) than practices (0.27/1000 AEs) [14].

The overall rate of SRC in high school soccer in the USA from 2005 to 2014 was estimated to be 0.36/1000 AEs (girls = 0.45/1000; boys = 0.28/1000 AEs). The rate of concussion in games versus practices was higher in girls when compared with boys [15].

Marar *et al*. reported a competition concussion rate of 0.92 per 1000 AEs among female high school soccer athletes, with a rate of 0.53 per 1000 AEs among male players [16]. Among youth and high school players, concussion is the second most common game injury representing approximately 24% of all injuries [17].

One of the earliest surveillance studies on concussions in college age male and female players reported the concussion rate to be 0.6/1000 AE for men and 0.4/1000 AE for women [18].

Data based on professional players come from both the domestic professional season and tournament settings. Concussions are the fifth most common injury in the US Major League Soccer, although injury rates vary by team and season [19]. Junge and Dvorak summarized the injury data collected during 51 FIFA-sponsored tournaments and four Olympic Games from 1998 to 2012, noting that 15% of injuries affected the head or neck [20].

### Mechanisms of injury

Mechanisms of head injury in soccer include unintentional hits to the head through contact with body parts of other players, against the ground, soccer goal frame or from the ball to an unprepared player [10,21,22]. Unique to soccer are the repetitive forces or subconcussive trauma involved in heading the ball [10,23,24]. Subconcussive head impact is defined as an impact to the head that does not induce clinical symptoms of concussion and has emerged as a complex public health issue. Rotational acceleration, linear acceleration and carotid artery injuries have been reported by researchers as having an impact on the development of neuropathological changes in acute brain trauma suffered in boxing as well as in soccer [21,25,26].

Player-to-player contact has been noted to be the mechanism responsible for the greatest proportion of concussions in both male and female soccer athletes [27]. One prospective study of collegiate female and male players over 2 years found that about 70% of concussions occurred during games, and that head-to-head contact was the most frequent mechanism of injury, followed by head to ground and head to other body parts. None of the concussions in this study resulted from intentional heading of the ball [18]. Additionally, the majority of concussions in youth and high school players occur when the player is unaware of the oncoming contact or collision [28]. The risk of concussive events is increased in games due to a greater quantity of head impacts and not necessarily greater severity [29].

In another retrospective study of high school and youth soccer players between 2005 and 2014, nearly 60% of concussions involved contact or collision with another player and the player behavior of heading the ball represented 28% of all concussions, 70% of which were from player-to-player contact, not ball-to-player contact [15].

Andersen *et al*. examined 192 head injury incidents involving player-to-player contact in Norwegian and Icelandic professional players and reported that 41% of concussions resulted from contact by an elbow, arm or hand to the head [9]. With respect to playing situation, 58.3% of head injuries were sustained while the player was engaged in a heading duel [9]. In a similar cohort of high school athletes, Marar *et al*. reported concussions accounted for 60.0% of all injuries sustained while heading the ball [16], while another study reported a proportion of 64.1% [30].

In an examination of 69 concussions in 60 collegiate athletes, Delaney *et al*. found that blows to the temporal region of the head were most likely to result in concussion for soccer [31]. Defenders and goalkeepers have also been reported to sustain more concussive injuries than forwards or midfielders [32–34].

### Sex differences

Among female athletes, concussions are reported most often among soccer players with concussion incidence as high as 1.9 per 1000 competition-related athlete-exposures and 0.6 per 1000 overall athlete-exposures [35–37]. This compares with 0.53 per 1000 competition-related athlete-exposures and 0.28 per 1000 overall AEs in males [15,16]. Chandran *et al.* examined sex differences in head injuries and concussions among collegiate soccer players between 2004 and 2009, finding a higher rate of head injuries in women than men with the rate of injury due to contact with an apparatus (ball/goal) nearly 2.5-times higher and the rate due to contact with a playing surface over two-times higher in women [38].

Sex differences in anthropometrics and heading kinematics have been examined among Division I soccer athletes, with women found to display lower anthropometry measures compared with men, resulting in increased head impact kinematics during soccer heading. Decreased neck girth and strength among female players were found to be potentially associated factors [39]. Similarly, in a study of controlled soccer headers, Caccese *et al.* found higher peak linear and rotational acceleration in females compared with males, suggesting that females may be exposed to greater traumatic shearing forces associated with brain injury [40].

Sex may also play a role in determining recovery after concussion, with female soccer players performing worse on neurocognitive testing and also reporting more symptoms than male soccer players [41]. Covassin *et al.* reported that female concussed soccer players conveyed more total concussion symptoms at 8 days compared with male concussed athletes [42]. In addition, significant effects for sex on verbal and visual memory were documented, with female athletes reporting lower scores and more symptoms on the migraine-cognitive-fatigue and sleep clusters, respectively [42].

In contrast, Zuckerman *et al*. examined 40 male and 40 female soccer players using the Immediate Postconcussion Assessment and Cognitive Testing, finding no difference based on sex in the acute response to concussion among high school soccer players [43].

### Biochemical & structural implications of RSHI & concussion in soccer

As previously discussed, subconcussive impact due to repetitive heading, acceleration, deceleration and rotational forces on the brain may also result in structural and functional changes. Such changes have been identified in both former and active soccer players, and studies have postulated that these neurochemical and neurostructural changes may occur before evidence of neurobehavioral change [44,45].

Using diffusion tensor imaging (DTI), Myer *et al.* described the ability of a specialized neck collar to dampen the effects of subconcussive impact on white matter integrity through bilateral jugular vein compression, diverting blood flow to vertebral veins and promoting venous engorgement. The authors reported pre- to postseason changes in mean diffusivity, axial diffusivity and radial diffusivity in a noncollar group versus collar group, finding significant correlation between head impact exposure and DTI changes over the season in the noncollar group [46].

In a study of 37 amateur soccer players, Lipton *et al.* studied the effects of repetitive heading on fractional anisotropy and cognitive function, and reported that heading was associated with lower fractional anisotropy at three locations in temporo-occipital white matter and that lower levels of fractional anisotropy were associated with poorer memory scores with a threshold of 1800 headings per year [47].

Magnetic resonance spectroscopy has also been used to examine the effects of RSHI on neurochemistry. Koerte *et al*. found significant increases in both choline (a membrane marker) and myo-inositol (a marker of glial activation) in 14 former professional soccer players without a history of clinically diagnosed concussion compared with control athletes [48].

Researchers have also reported electroencephalographic abnormalities and protracted postconcussive symptoms in a group of 37 former Norwegian national team players [49–51].

Other technologies, including transcranial sonographic measurement of optic nerve sheath diameter (a noninvasive technique to predict raised intracranial pressure), and various cerebrovascular biomarkers have been proposed to assess the effects of RSHI in soccer players [52,53]. Sadrameli *et al*. observed an increase in optic nerve sheath diameter that was independent of concussions in 24 female collegiate soccer players [52].

Several cerebrospinal fluid (CSF) biomarkers for RSHI and concussion have also been analyzed in former and active soccer players. Biomarkers such as neurofilament light protein, glial fibrillary acidic protein and S-100B have provided insight into the pathophysiological mechanisms of TBI and are indicators of neuronal, axonal and astroglial damage resulting from TBI [54].

Zetterberg *et al*. investigated the effects of repetitive heading on CSF concentrations of neurofilament light protein, total tau, glial fibrillary acidic protein, S-100B and albumin, with the results showing no correlation between the CSF biomarkers and number of headers performed. Dorminy *et al*. found no correlation between S-100B, concussion assessment test scores and repetitive heading in 16 division 1 soccer players across a range of ball velocities [55]. Conversely, Mussack *et al.* observed S-100B levels of a controlled amateur heading group, and reported transient increases 60–360 min post-training that were significantly elevated compared with normal exercise [56]. Wallace *et al.* also found elevated levels of neurofilament light chain at 1 h and 1 month following a repetitive heading session, as well as elevated total symptoms and symptom severity on the SCAT3 test [57]. These results suggest heading may lead to biochemical signs of axonal damage in serum, at least in the short term. Larger-scale studies that correlate serum biomarker findings with long-term neuropsychological development and clinical symptoms are warranted.

### Soccer & neurodegenerative disease

Data regarding incidence and prevalence of CTE pathology and neurodegenerative disease in soccer are limited due to the small number of case studies and the retrospective nature of brain bank specimen analysis [45,58–62]. Koerte *et al.* identified greater cortical thinning with age in 15 male former professional soccer players, specifically in the right inferolateral–parietal, temporal and occipital cortex compared with controls [63].

A recent study tracked 14 retired soccer players with dementia from 1980 to 2010 and reported neuropathologic findings of septal abnormalities in all six postmortem cases, supportive of a history of chronic repetitive head impacts. Four of the cases had pathologically confirmed CTE [45].

Mackay *et al*. conducted a recent retrospective cohort study comparing mortality from any neurodegenerative disease among 7676 former professional Scottish soccer players with 23,028 general population controls. They found that mortality from neurodegenerative disease was higher (1.7 vs 0.5%) and mortality from other common diseases lower in the soccer players compared with matched controls [64].

The Boston University CTE Center and Brain Bank (MA, USA) has published several studies examining CTE, with the majority of cases including former boxers (85%), although also including cases from former American football players, soccer players and wrestlers [65]. The center recently announced a new study on Soccer, Head Impacts and Neurological Effects that is enrolling 20 former female professional soccer players to study the long-term cognitive effects of RSHI [66].

At present, no data beyond autopsy studies exist to support the hypothesis that soccer participation is a risk factor for the development of neurodegenerative disease like CTE.

### Neuropsychological implications of RSHI & concussion in soccer

Recent research suggests that exposure to both concussive and subconcussive events can result in persistent cognitive and neuropsychologic impairments [67]. For soccer, studies examining subconcussive impacts have demonstrated no effects of heading on cognitive function after a 15-min heading session [19], after two practices or games, after one season and through assessment with a cross-sectional analysis [68]. Forbes *et al*. found no association between neuropsychological test performance and concussive impacts in soccer [69], while others have demonstrated no significant relationship between computerized neurocognitive test performance and an athlete’s exposure to increased concussive and subconcussive impacts through soccer [70,71]. Conversely, data from Di Vigilio *et al*. using transcranial magnetic stimulation suggested changes in cognitive performance and increased intracortical inhibition following a single exposure to subconcussive head impacts from routine soccer heading [72]. These results echo those found by Webbe *et al*. in which ball-to-head contact was associated with at least transient cognitive impairment [73]. Levitch *et al*. demonstrated that soccer RSHI affected neurophysiologic function differentially based on the chronicity of exposure, with recent heading impacting psychomotor tasks and long-term heading exposure affecting verbal memory and learning [74].

Overall, studies investigating the neuropsychological implications of RSHI and concussion in soccer are lacking due to their retrospective nature and low number of subjects [19,67–77]. The limited available data do not support the hypothesis that there are intermediate or long-term adverse neurocognitive effects from heading the ball in soccer.

### Injury prevention

Recognition and awareness of concussions through external observation and removal of athletes from play is of paramount importance. Educational resources have been published by many organizations, including the Center for Disease Control and Prevention, the National Collegiate Athletic Association (IN, USA), US Soccer (IL, USA) and the United Soccer Coaches (MO, USA) [78]. Current guidelines, including those from FIFA, state that athletes diagnosed with SRC should be removed from play and evaluated by a healthcare provider trained in concussion management, and should not return to play the same day [79]. An initial period of cognitive and physical rest is recommended [80]. Return to academic and sport process then includes a stepwise symptom-limited progression of increased physical, cognitive, work or school-related activity [79].

Unfortunately, as many as 44% of elite female youth soccer players in one study indicated that they would not report their concussion symptoms [81], while another study of elite female soccer players aged 12–15 demonstrated that the majority (59%) of concussed players continued to play with concussive symptoms [82]. The use of in-game spotters who can review live game film or gameplay to identify indicators of possible concussion may be beneficial; however, costs and logistics of implementation are not feasible for most nonprofessional/elite organizations.

Currently, US Soccer’s policy on age-related heading prohibits heading for 10 years old and under, with ages 11–12 limited to a maximum of 30 min of heading training per week [40,83,84]. The danger of repetitive heading in soccer has been called into question multiple times previously with several groups more recently calling for a ban on heading among soccer players younger than 14 years of age [5,85,86]. While these efforts have served to decrease the number of head-to-ball contacts in practices and games, contact with another player has consistently shown to be the most common mechanism of injury in heading-related concussions among boys and girls [10,15,22,62,77]. Thus, reducing athlete contact through rule changes or stricter enforcement may be a more effective way to prevent concussions and other injuries [15]. Stricter rules punishing aerial challenges that involve elbows to the head, head-to-head or hand-to-head contact (e.g., goalkeeper) may diminish rates of head injury. Andersen *et al*. reported that strict enforcement and interpretation of the laws of the game was associated with a lower incidence of head injury caused by player-to-player contact [9].

The role of headgear for injury prevention has been examined with results demonstrating no reduction in impact accelerations or the incidence and severity of SRC in soccer [87,88]. Concerns also exist that universal use of headgear might lead to more aggressive heading and head challenges, resulting in a paradoxical increased risk of injury [89]. In contrast, Delaney *et al*. reported that female adolescent soccer players who did not wear headgear were more susceptible to concussion [90].

Educating players on the biomechanics of heading as well as strength training and conditioning may be other important areas of focus for injury prevention. Behavioral skills training for heading has previously been described [91]. Babbs *et al*. examined the safety of heading a soccer ball by calculating head accelerations, reporting that heading is usually safe, although occasionally dangerous, depending on characteristics of the player and ball. Low head–neck segment mass predisposes athletes to higher head acceleration. Head–neck–torso alignment during heading and follow-through after contact can be used to decrease head acceleration [92]. Safety is also improved when players head the ball with greater effective body mass. Thus, younger players with smaller bodies are at risk of potentially more dangerous headers. As discussed, there are sex differences in heading kinematics, with women exposed to higher rotational head acceleration and resultant shearing forces due to relative neck weakness compared with men. Strength and awareness training may increase an athlete’s ability to prepare and brace for an impact to the body or head and is an important area of future research [93]. The use of lower ball inflation pressures, teaching of proper heading technique and redesign of age-appropriate balls for young players may also mitigate the risks of dangerous head accelerations [94].

More recently, specialized technologies have been developed to both record AE to head impacts and to diminish the consequences of these impacts. The earlier discussed specialized neck collar, designed to reduce intracranial energy absorption, has been shown to preserve white matter integrity in studies of football and hockey athletes [95,96]. In female high school soccer athletes, Myer *et al*. found microstructural changes in the white matter tracts on DTI in athletes who did not wear the collar device in comparison to athletes who wore the device [46]. Correlation of these imaging changes with neurocognitive outcomes is unclear.

In an attempt to better quantify head impact exposures in soccer, Press *et al*. used linear accelerometer sensors worn on the mastoid and compared impact exposures recorded by the devices to exposures recorded via video. They found that the sensors reported a much greater total number of impacts than that identified through video analysis [97]. Improvements in sensitivity and specificity of monitoring technologies may eventually lead to earlier identification of potential concussive head impacts during competitions.

## Discussion

Soccer-related concussion represents a significant proportion of SRC worldwide. While sports such as hockey and American football have received substantial attention and research in regards to concussion, studies exclusively examining soccer are lacking.

After review of the soccer concussion literature, studies suggest that women experience a greater rate of concussion in both practices and games compared with men, and that female soccer players have the highest rates of concussion when compared with female athletes in other sports. Concussions in soccer most commonly occur due to player-to-player contact rather than ball-to-head contact, with purposeful heading rarely resulting in concussion. The majority of studies examining the biochemical, structural and cognitive implications of RSHI and concussion in soccer have included only small numbers of athletes. At present, no data support the hypothesis that soccer participation is a risk factor for the development of CTE, though CTE has been found in small autopsy studies of former professional soccer players. Further large-scale studies are warranted.

While there have already been efforts to decrease the prevalence of soccer-related concussion through the use of headgear, limitations on heading, strength training and player education, it is unclear how these efforts have impacted concussion rates. Future attempts to quantify concussion frequency and severity more accurately with mastoid sensors, spotters and other novel technologies will lead to a greater understanding and ultimately ability to prevent concussions in soccer. For example, limiting athlete contact during soccer practices and games may have a greater impact on concussion rate than limiting heading of the ball. Future studies examining the effects of preventative interventions are warranted.

Limitations of this study include the low number of high-level studies included with only one randomized control trail (Table 1), as well as the small number of athletes included in these studies. Additionally, a large proportion of the studies represented only North American soccer players, highlighting a lack of high-level and large-scale international studies. While using the search term soccer instead of football may have limited the proportion of international studies, using the term football resulted in many nonrelevant articles solely examining American Football.

## Conclusion

Given the lack of high-level research examining SRC and RSHI in soccer, as well as the popularity and growing number of athletes who play soccer around the world, scientists and clinicians should focus their efforts to better understand the specific epidemiology, pathophysiology, injury biomechanics, as well as the short- and long-term consequences of soccer-related concussion. This more nuanced understanding will be necessary to formulate and institute effective interventions that will make the sport safer for its participants.

## Future perspective

In the near future, with ongoing research and technological improvements, electronic sensors and spotters may acquire the sensitivity and specificity to detect concussive impacts better than humans, resulting in prompt removal from play of affected individuals for evaluation by medical personnel.

Studies seeking to identify blood and even salivary markers for mild TBI and SRC are promising and expanding. One could envision a future where a suspected concussed athlete is diagnosed on the sideline using an objective and validated blood or salivary biomarker.

Additionally, stricter enforcement of existing rules and new rules limiting player-to-player contact, particularly aerial challenges where head impact risk is greatest, will serve to diminish concussion risk in soccer in the future. Age- and gender-specific research and recommendations will also serve to protect those populations most at risk. While American football and hockey have been subjected to rigorous and focused scientific attention in regard to concussion, the research examining soccer-related concussion remains in its infancy. Future large-scale basic scientific and prospective studies will expose the true consequences of RSHI and concussion in soccer and reveal new avenues for improvements in the safety of the game.

**Executive summary**

- The majority of soccer-related injuries, including head injuries and concussions, occur among those under the age of 25.
- Concussions are more likely to occur in soccer games than in practices and have a higher incidence in female versus male athletes.
- Player-to-player contact is the most common mechanism resulting in concussions in both male and female soccer athletes.
- Purposeful heading rarely results in concussion.
- Female soccer players have the highest concussion rate of all female athletes and may have a prolonged recovery compared with male soccer athletes.
- Imaging changes on diffusion tensor imaging, magnetic resonance spectroscopy, transcranial sonography and electroencephalographic have been noted in active and former soccer players.
- Studies examining biomarkers for concussion in soccer have yielded mixed results with some reporting elevated S-100B and neurofilament light chain after soccer RSHI.
- No data at present support the hypothesis that soccer is a risk factor for development of neurodegenerative diseases.
- Studies are lacking but do not demonstrate intermediate or long-term adverse neurocognitive effects from heading the ball in soccer.
- Recognition and awareness of concussions during soccer play and removal of those affected is of paramount importance and officials should be trained to recognize signs and symptoms with referral to appropriate medical care. Technologies designed to identify potential concussive impacts will prove beneficial once refined.
- Reduction of player contact along with biomechanical/behavioral education, strength/awareness training and restructuring/enforcement of rules will all serve to reduce concussion incidence in soccer.
